# Liquid Biopsy Biomarkers for Cervical Cancer: A Systematic Review

**DOI:** 10.3390/ijms262110503

**Published:** 2025-10-29

**Authors:** Jesús Alejandro Pineda-Migranas, Juan Carlos Bravata-Alcántara, Iliana Alejandra Cortés-Ortíz, Enoc Mariano Cortés-Malagón, María de los Ángeles Romero-Tlalolini, Mónica Sierra-Martínez, Gustavo Acosta-Altamirano

**Affiliations:** 1 Escuela Superior de Medicina, Instituto Politécnico Nacional (IPN), Salvador Díaz Mirón esq. Plan de San Luis y Díaz Mirón s/n, Col. Casco de Santo Tomás, Mexico City 11340, Mexico; jesuspm23@yahoo.com.mx; 2 Health Research Unit, Hospital de Alta Especialidad Ixtapaluca, Servicios de Salud del Instituto Mexicano del Seguro Social para el Bienestar (IMSS-BIENESTAR), Carr Federal México-Puebla Km 34.5, Ixtapaluca 56530, Mexico; juan.bravata@hraei.gob.mx; 3 Facultad de Medicina y Cirugía, Universidad Autónoma “Benito Juárez” de Oaxaca (UABJO), Oaxaca 68020, Mexico; romerotlalolini@gmail.com; 4 Molecular Genetics and Diagnostic Laboratory, Hospital Juárez de México, Instituto Politécnico Nacional, 5160, Col. Magdalena de las Salinas, Mexico City 07760, Mexico; iliancortes@yahoo.com.mx; 5 Research Division, Hospital Juárez de México, Instituto Politécnico Nacional, 5160, Col. Magdalena de las Salinas, Mexico City 07760, Mexico; emcortes@cinvestav.mx; 6 Hospital General de México “Dr. Eduardo Liceaga”, Eje 2A Sur (Dr. Balmis) No. 148, Cuauhtémoc, Doctores, Mexico City 06726, Mexico

**Keywords:** cervical cancer, liquid biopsy, circulating microRNAs, cfHPV-DNA, serum cytokines, biomarkers, molecular diagnostics

## Abstract

Cervical cancer remains a significant public health priority, particularly in low- and middle-income countries. In this context, liquid biopsy has emerged as a minimally invasive method for detecting and monitoring molecular biomarkers, offering advantages over traditional screening approaches. This systematic review included 21 studies published between 2015 and 2025 and was conducted in accordance with the PRISMA 2020 statement. The analysis examined the role of serum cytokines, circulating microRNAs (miRNAs), and circulating cell-free HPV DNA (cfHPV-DNA) in patients with cervical cancer or high-grade intraepithelial lesions. Circulating miRNAs—particularly miR-21, miR-29a, and miR-34a—are consistently associated with recurrence, tumor progression, and reduced survival. However, their immediate clinical translation remains limited by methodological variability and the lack of universal normalizers. In contrast, cfHPV-DNA, especially with ddPCR, exhibited the best study-level performance, with a specificity of 100% and a sensitivity of approximately 80–88%, across heterogeneous endpoints and analytic conditions. Consequently, cfHPV-DNA represents a promising tool for post-treatment surveillance and early detection of recurrence. Serum cytokines, such as TNF-α, IL-6, and IL-10, reflect inflammation and the tumor microenvironment. Nevertheless, their lack of standardization and variability across detection platforms restricts their reproducibility, positioning them as complementary rather than stand-alone markers. In conclusion, the evidence supports liquid biopsy as a promising tool in cervical cancer management; nonetheless, only cfHPV-DNA is currently ready for clinical application, whereas miRNAs and cytokines require multicenter validation and technical standardization before implementation.

## 1. Introduction

In 2025, cervical cancer remains one of the most common causes of female mortality, particularly in low- and middle-income countries. The structural inequity associated with this disease is evident, as more than 80% of related deaths occur in resource-limited regions [1,2]. Globally, an estimated 660,000 new cases and 348,000 deaths are reported annually. In Mexico, approximately 10,348 new cases and 4909 deaths were registered in 2022. According to Cruz-Valdez et al. [3], cervical cancer is the second leading cause of gynecological death after breast cancer.

Persistent infection with high-risk HPV is a necessary but not sufficient condition for cervical carcinogenesis. Once viral genome integration occurs, the host’s genetic regulation undergoes irreversible alterations. Dysregulation of microRNAs (miRNAs) further complicates the processes of differentiation, cell proliferation, and apoptosis. Similarly, abnormal secretion of cytokines generates an immunosuppressive and proinflammatory tumor microenvironment that favors cancer progression [4,5,6].

Mortality rates have declined significantly in developed nations due to the implementation of modern screening methods such as cytology and HPV DNA testing. However, significant limitations persist in fragile health systems: cytology remains operator-dependent with <60% sensitivity in early stages, whereas HPV molecular assays fail to distinguish between transient and persistent infections. This leads to patient anxiety, overdiagnosis, and additional strain on already overburdened health systems [7,8,9].

Liquid biopsy is a minimally invasive approach that detects tumor-derived components—such as circulating tumor cells, circulating tumor DNA, coding and noncoding RNAs, and extracellular vesicles—in blood and other biofluids, enabling real-time assessment of tumor heterogeneity, treatment response, and minimal residual disease and permitting repeated sampling for longitudinal monitoring. Over the past decade, liquid biopsy has emerged as a powerful tool in oncology, enabling the detection of biomarkers in body fluids—primarily peripheral blood—in a dynamic and minimally invasive manner. The most widely studied biomarkers in cervical cancer are circulating miRNAs, which reflect early epigenetic alterations; circulating HPV DNA (cfHPV-DNA), which is directly associated with tumor burden and treatment response; and serum cytokine profiles, which provide insights into systemic inflammation and the tumor immune microenvironment [10,11,12,13,14].

Preliminary studies in Mexico and Latin America have tested the feasibility of these biomarkers. González-Ramírez et al. [15] reported that miRNA signatures are associated with prognosis and disease progression. In addition, the incorporation of self-collected cervicovaginal samples into HPV-based screening programs has been explored [16,17], and cfHPV-DNA has been shown to predict relapse [11]. However, their immediate clinical use is limited by small sample sizes and methodological variability. Systematic evaluations that integrate both regional and international evidence are therefore essential.

This review aims to analyze and compare the most recent scientific evidence on cfHPV-DNA, serum cytokines, and circulating miRNAs in liquid biopsy samples to determine their potential as biomarkers of cervical cancer. Furthermore, it explores their feasibility and clinical applicability for integration into public health programs, not only in Mexico but also across Latin America.

## 2. Methods

### 2.1. Protocol and Register

Following the recommendations of the PRISMA 2020 statement [18], this study was designed and conducted as a systematic review. To ensure transparency, prevent duplication, and strengthen methodological validity, the review protocol was registered in PROSPERO (International Prospective Register of Systematic Reviews) with the registration number CRD420251150875. The PRISMA 2020 checklist and PROSPERO registration are provided as Supplementary (nonpublished) Materials.

### 2.2. Eligibility Criteria

We included original articles published between January 2015 and May 2025 that examined the role of liquid biopsy biomarkers—such as serum cytokines, circulating microRNAs (miRNAs), and circulating cell-free HPV DNA (cfHPV-DNA)—in individuals with confirmed cervical cancer or high-grade intraepithelial lesions. Both clinical trials and observational studies were eligible if they provided data on sensitivity, specificity, predictive values, or clinical correlations. To ensure clinical applicability, editorial reviews, narrative articles, letters to the editor, conference abstracts, and studies conducted in cell lines or animal models were excluded [18].

### 2.3. Information Sources and Search Strategy

To ensure comprehensiveness and reproducibility [19,20], the search strategy was developed in accordance with the Cochrane Handbook for Systematic Reviews of Interventions and PRESS (Peer Review of Electronic Search Strategies) guidelines. We searched the LILACS, Web of Science, Embase, Scopus, and PubMed/MEDLINE databases for articles published between January 2015 and May 2025. Keywords and MeSH terms—both free-text and controlled vocabulary—were used in combination with the Boolean operators “AND” and “OR.” The strategy included terms related to “cervical cancer,” “liquid biopsy,” “circulating microRNAs,” “cfHPV-DNA,” and “serum cytokines.” See Supplementary Materials (S1).

### 2.4. Study Selection

All records identified through the database searches were exported to a reference manager to remove duplicates. Two independent reviewers subsequently applied the inclusion and exclusion criteria to screen titles and abstracts. Potentially eligible full-text articles were retrieved and assessed for relevance. Discrepancies were resolved through consensus and, when necessary, by consulting a third reviewer.

Across the databases reviewed, the systematic search identified 482 records. After 112 duplicates were removed, 370 titles and abstracts were screened. Among these studies, 310 did not meet the eligibility criteria and were excluded because they were narrative reviews, case reports, or studies conducted in animal models. The full texts of the remaining 60 articles were assessed. After applying the inclusion criteria strictly, 21 studies were selected, all of which provided direct clinical evidence on the use of liquid biopsy biomarkers in patients with cervical cancer.

**Figure 1:**
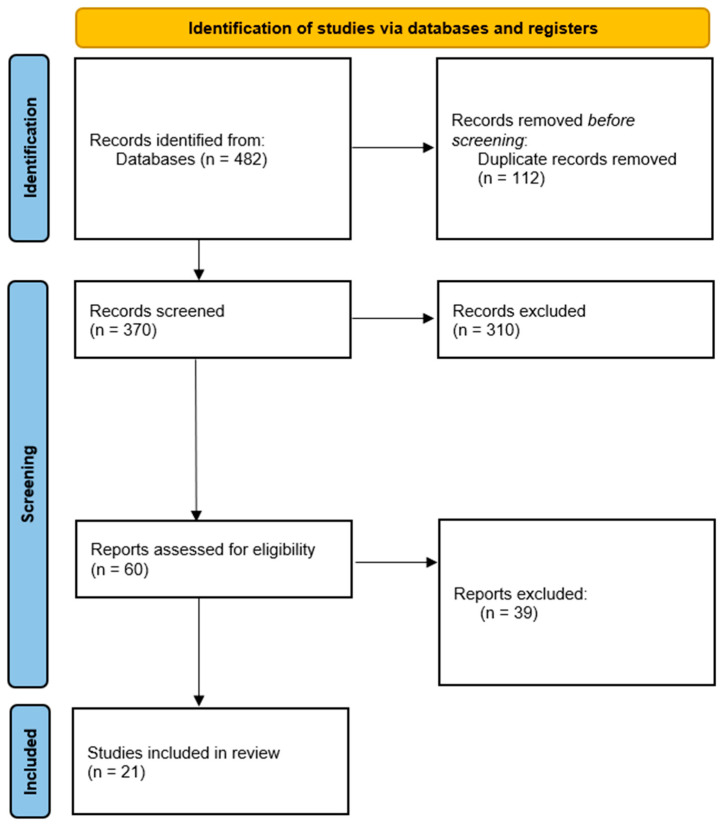
PRISMA 2020 flow diagram for the selection of studies included in the systematic review. Of the 482 records initially identified, 112 duplicates were removed. After screening 370 titles and abstracts, 310 were excluded because they did not meet the eligibility criteria. The remaining 60 full-text articles were assessed, and 21 studies ultimately met the inclusion criteria and were included in the qualitative synthesis.

The selection process is summarized in the PRISMA 2020 flow diagram (Figure 1).

### 2.5. Data Extraction Process

Information from the included studies was collected via a standardized form that incorporated the following variables: author and year of publication, country, methodological design, sample size, type of biomarker analyzed, detection platform employed (RT-qPCR, ddPCR, NGS, ELISA, and others), clinical outcomes, and main findings. Data extraction was performed independently by two reviewers, and to minimize bias, the results were consolidated into a single database.

### 2.6. Risk of Bias Assessment

Validated tools were applied in accordance with the study design to evaluate methodological quality. The ROBINS-I tool [21] was used for observational studies. The RoB 2 tool [22] was employed for randomized clinical trials. AMSTAR-2 [23] was applied to systematic reviews included as methodological references. All assessments were conducted independently by two reviewers, and disagreements were resolved through consensus.

The risk of bias was assessed with ROBINS-I (Version 2) across seven domains, with study-level judgments (low/moderate/severe/critical) and an algorithm-derived overall rating. No randomized trials were included; RoB 2 was not applicable. The results are presented as traffic-light and domain-summary plots (Supplementary Materials, S2).

### 2.7. Synthesis Methods

A qualitative narrative synthesis was selected because of the diversity of study designs, populations, and detection platforms. The findings were structured into three main categories: cfHPV-DNA, serum cytokines, and circulating microRNAs. Limitations, trends, and discrepancies among the studies were critically described without conducting a meta-analysis, as this was not planned in the protocol and was methodologically unfeasible given the variability in the results. To guide this stage, the PRISMA-DTA guidelines [24] were considered, along with recent reviews of circulating biomarkers in cervical cancer as contextual references [25].

## 3. Results and Discussion

### 3.1. General Characteristics of the Studies

Table 1 summarizes the 21 studies that investigated liquid biopsy biomarkers related to cervical cancer. These studies revealed considerable variability in clinical, geographic, and methodological contexts. Most were conducted in Europe (*n* = 10) and Asia (*n* = 8), while only three came from Latin America: one from Mexico [26] and two from Brazil [11,27]. The sample sizes ranged from 35 to more than 400 patients, which influenced both the statistical power and the generalizability of the findings. Prospective observational studies predominated, although some preliminary clinical trials were also reported, primarily aimed at validating detection platforms.

With respect to methodologies, ddPCR [34,40] has been widely used to investigate cfHPV-DNA, whereas RT-qPCR has been used predominantly to quantify microRNAs [26]. Serum cytokine profiles were characterized via multiplex immunoassays and ELISA [27,42,43,44]. Given the heterogeneity of outcomes and parameters, it is more appropriate to present the findings qualitatively rather than through a meta-analysis.

Three studies provided direct evidence in the Latin American context. In Mexico, Zubillaga-Guerrero et al. [26] reported the overexpression of miR-16-1 by liquid-based cytology, which was associated with high-risk HPV integration. In Brazil, Bonin-Jacob et al. [27] reported that elevated serum levels of IL-6 and IL-10 were correlated with advanced disease stages. Additionally, the persistence of plasma cfHPV-DNA after therapy was shown to indicate recurrence and a poorer survival prognosis [11]. Collectively, these studies highlight regional interest in validating replicable, accessible biomarkers, particularly in resource-limited settings.

On the other hand, studies evaluating serum cytokines are the most heterogeneous, mainly because of the lack of universal cutoff values and the variability in measurement platforms (primarily ELISA and multiplex assays). This methodological inconsistency affects the reproducibility of findings and complicates cross-study comparisons. Taken together, these results suggest that although the body of evidence is increasing, multicenter standardized protocols are still needed to reduce heterogeneity and strengthen clinical validity [23].

### 3.2. Main Findings by Biomarker

Research on circulating microRNAs revealed that profiles such as miR-21, miR-29a, and miR-34a [33,45] are consistently associated with advanced tumor progression, reduced overall survival, and early recurrence. The clinical standardization of their application is limited by variability across detection platforms (RT-qPCR, ddPCR, NGS) and the absence of universally accepted normalization methods. However, some individual studies have reported sensitivities and specificities exceeding 90% [45].

Most studies have concluded that cfHPV-DNA is a reliable indicator of tumor burden and a strong predictor of post-treatment recurrence. In Asian and European cohorts, persistent cfHPV-DNA following chemoradiotherapy was associated with early relapse and shorter overall survival. Among studies reporting diagnostic discrimination, study-level sensitivities typically range from ~80–88%, and specificities frequently approach 100%, particularly with ddPCR [10,35,36]. A recent investigation from Brazil [11] corroborated its predictive value in the Latin American setting. Collectively, these findings confirm that cfHPV-DNA is the most robust biomarker with the most significant potential for immediate clinical application.

In terms of serum cytokines, TNF-α, IL-10, and IL-6 are associated with advanced disease stage and a poor prognosis [27,42,43,44]. However, the absence of standardized procedures and reference cutoff values limits the strength of these conclusions. Although cytokines cannot be used as standalone diagnostic tools, they function as complementary biomarkers that help characterize the tumor microenvironment and chronic inflammation [27,42,43,44].

**Figure 2:**
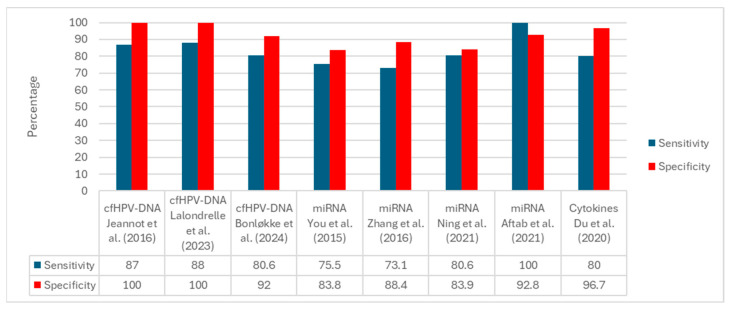
Prestudy sensitivity and specificity by biomarker family. Bars display single-study estimates organized as cfHPV-DNA, miRNA, and cytokine panels. Substantial between-study heterogeneity is evident in clinical endpoints (e.g., diagnostic discrimination, baseline cfHPV-DNA reference to tissue HPV, treatment-decision classification) and methodological features (e.g., matrix, assay platform, analytical thresholds, reference standards). The cytokine example corresponds to a combined protein-plus-miRNA panel rather than cytokines alone [29,30,31,32,33,34,38,39].

Figure 2 displays the per-study sensitivity and specificity grouped by biomarker family. Among studies reporting both metrics, cfHPV-DNA assays show the highest performance, with specificity frequently reaching 100% and sensitivity typically in the ~80–88% range. miRNA panels exhibit intermediate performance with wider dispersion (sensitivity ~73–100%, specificity ~83–93%) depending on the assay, matrix, and cutoff. Cytokine-only investigations seldom report paired sensitivity/specificity; the example shown corresponds to a combined protein-plus-miRNA panel with high specificity (~96.7%) and sensitivity of approximately 80%. Overall, the comparative pattern favors cfHPV-DNA for diagnostic discrimination, whereas miRNAs appear promising but method dependent, and cytokines are primarily supported by prognostic/associative evidence.

### 3.3. Narrative and Quantitative Synthesis

Among the 21 included studies, fifteen provided data for narrative synthesis, and 8 provided data for exploratory quantitative analyses. The evidence demonstrates that circulating miRNAs are valuable diagnostic and prognostic tools, but methodological variability remains the main limitation for their use in clinical practice. Differences in extraction methods, detection platforms, and the absence of universal normalizers account for the heterogeneity observed, which in turn compromises reproducibility. Although profiles such as those of miR-21, miR-29a, and miR-34a have been consistently associated with tumor progression and reduced overall survival, these findings require confirmation in extensive multicenter studies before their clinical application can be considered.

In contrast, cfHPV-DNA yielded more consistent findings. Across European, Asian, and Latin American cohorts, on-treatment or post chemoradiotherapy persistence of cfHPV-DNA was associated with early recurrence and poorer prognosis. Among studies reporting diagnostic discrimination, study-level sensitivities typically range from approximately 80–88%, and specificities frequently approach 100%, particularly with ddPCR, despite heterogeneity in endpoints, matrices, and analytical thresholds. These findings confirm cfHPV-DNA as the most robust biomarker and the only one already close to clinical application, in line with the global trend of integrating molecular biomarkers into oncology follow-up algorithms.

Although serum cytokines are reliable indicators of immune status and chronic inflammation, their immediate clinical utility remains limited because of the lack of standardized cutoff values and wide variability across detection platforms. Studies linking TNF-α, IL-10, and IL-6 with advanced disease and poorer overall survival support their use as complementary biomarkers, mainly for characterizing the tumor microenvironment rather than as standalone diagnostic tools.

Overall, liquid biopsy represents a promising approach for the comprehensive management of cervical cancer, as confirmed by the data summarized in Table 1 and the comparative estimates shown in Figure 2. However, only cfHPV-DNA is currently ready for immediate clinical use. In contrast, circulating miRNAs and serum cytokines still require multicenter protocols, standardized validation, and international consensus before being recommended for national screening or follow-up programs.

### 3.4. Discussion

This systematic review revealed that liquid biopsy biomarkers, including circulating microRNAs, cfHPV-DNA, and serum cytokines, represent promising options for improving the diagnosis, surveillance, and prognosis of patients with cervical cancer. In clinical practice, their use could help overcome the limitations of traditional screening tests, particularly in settings with high disease burdens and fragile healthcare systems.

Circulating microRNAs are the most frequently studied biomarkers. Profiles such as miR-21, miR-29a, and miR-34a have been consistently associated with tumor recurrence, disease progression, and reduced overall survival [15]. Although study-level sensitivity and specificity vary across panels, matrices, and analytical cutoffs, the overall pattern indicates moderate diagnostic discrimination with notable heterogeneity. These observations are concordant with prior systematic reviews that likewise reported intermediate accuracy and emphasized the need for multicenter validation and technical standardization [45]. However, reproducibility remains hampered by the lack of universal normalizers, differences in detection platforms (NGS, ddPCR, RT-qPCR), and heterogeneity in sample types. Therefore, microRNAs should be considered biomarkers with high diagnostic and prognostic potential, but they still require multicenter validation and technical standardization before routine clinical application.

Another aspect to consider is the variability in the reference biomarkers. For example, the use of different internal controls for RT-qPCR in microRNA studies, as highlighted by Kepsha et al. [13], significantly impacts data reliability, making comparisons across studies difficult. In contrast, cfHPV-DNA has emerged as the most robust and reliable biomarker. Its persistence after chemoradiotherapy has been shown to predict early recurrence and poorer overall survival in Asian and European cohorts [35,36], and concordant signals across diverse cohorts and platforms, particularly when quantified by ddPCR, support its use for response assessment and minimal residual disease detection, with a clear specificity advantage over miRNA- and cytokine-based approaches. Moreover, a Scandinavian study demonstrated that plasma cfHPV-DNA levels correlate with tumor burden and clinical stage, further confirming its utility for monitoring patients with advanced disease [38]. According to the most recent international guidelines, these findings position cfHPV-DNA as the only liquid biopsy biomarker with immediate clinical feasibility. Its rapid clearance from the bloodstream post-treatment allows it to serve as a real-time monitor of therapeutic response, with several studies showing a significant decrease in cfHPV-DNA copy numbers following successful chemoradiation or surgery. This dynamic response makes it a powerful tool for identifying patients with minimal residual disease who might benefit from intensified adjuvant therapies, a finding increasingly supported by ongoing prospective clinical trials. [42]

Serum cytokines, on the other hand, provide more heterogeneous evidence. While some studies have reported that elevated TNF-α, IL-10, and IL-6 levels are associated with advanced disease and a poorer prognosis [27,43], others have shown that combinations such as the IL-10/IL-6/TNF-α panel can improve risk stratification [42]. Nevertheless, variability among platforms (ELISA, multiplex, and flow cytometry) and the absence of universal cutoff values limit the reproducibility of these results. Consequently, cytokines should be regarded as complementary biomarkers that provide insight into the tumor microenvironment and chronic inflammation but lack sufficient diagnostic power for independent use in clinical practice.

In contrast to international guidelines, the World Health Organization (2020) emphasized the importance of early detection and follow-up. Nevertheless, specific biomarkers are not yet recommended owing to a lack of multicenter evidence. To date, neither the American Society of Clinical Oncology (ASCO) nor the National Comprehensive Cancer Network (NCCN) has incorporated cfHPV-DNA into its official clinical guidelines. However, several prospective clinical studies have demonstrated its value as a predictor of recurrence and a surveillance tool [35,36], suggesting that it should be regarded as an innovative biomarker with high potential for future integration into international recommendations. Additionally, the feasibility of noninvasive sampling methods, such as self-collected cervicovaginal swabs, further supports the potential for integrating molecular screening into public health programs, especially in underserved regions where conventional cytology is limited.

Although evidence from Latin America remains scarce, the available data are encouraging. In Mexico, Zubillaga-Guerrero et al. [26] demonstrated an association between high-risk HPV infection and the overexpression of miR-16-1. Additionally, Okunade et al. confirmed the feasibility of self-collected cervicovaginal samples for molecular screening [8]. These local contributions, albeit preliminary, underscore the need for multicenter studies with larger sample sizes and standardized methodologies to validate and strengthen the current findings.

In summary, this review supports liquid biopsy as a complementary approach to conventional testing in patients with cervical cancer. However, current evidence suggests that only cfHPV-DNA has the robustness required for immediate clinical application, particularly for post-treatment surveillance and early recurrence detection. In contrast, circulating microRNAs and serum cytokines still require multicenter confirmation, technical standardization, and international consensus before they can be recommended for national screening and surveillance programs.

## 4. Conclusions

This systematic review concludes that liquid biopsy biomarkers hold great potential for optimizing the comprehensive management of cervical cancer. Among them, cfHPV-DNA, particularly when quantified by ddPCR, shows the most consistent, disease-specific performance across settings. Clinical translation now depends on harmonized thresholds and pre-analytical standardization (matrix handling, assay design, genotype coverage), together with prospective, decision-oriented validation to define how cfHPV-DNA can inform risk-stratified follow-up or treatment adaptation. In the near term, the evidence supports targeted, context-specific implementation for post-treatment monitoring and early identification of recurrence, with appropriate quality assurance and predefined clinical action pathways.

The clinical use of circulating microRNAs (such as miR-34a, miR-21, and miR-29a) remains limited because of the absence of standardized normalization methods and variability across platforms, despite their strong diagnostic and prognostic potential. Similarly, while serum cytokines reflect the tumor microenvironment and chronic inflammation, they lack the diagnostic strength to be used independently.

In Mexico and Latin America, strategies to implement these biomarkers in reference hospitals validate their use through multicenter studies and achieve technical standardization are urgently needed. The gradual incorporation of cfHPV-DNA, followed by the integration of microRNA and cytokine panels into national clinical guidelines, could increase diagnostic equity and help reduce the burden of this neoplasm in vulnerable populations.

Future research should focus on validating these biomarker panels in large, diverse cohorts, standardizing laboratory protocols, and evaluating their cost effectiveness in different healthcare systems. By establishing a clear, evidence-based pathway for their integration, liquid biopsy biomarkers can move from promising research tools to essential components of a comprehensive cancer management strategy.

## Figures and Tables

**Table 1:** Characteristics of the studies included in the systematic review on liquid biopsy biomarkers for cervical cancer ^1^.

#	Author (Year)	Country	Design	n	Sample	Biomarker	Platform	Main Findings	References
1	Ma et al. (2019)	China	Prospective	184	Plasma	miRNAs	RT-qPCR	A 5-miRNA signature discriminates against CC vs. controls	[28]
2	Du et al. (2020)	China	Prospective	260	Serum	Proteins + miRNAs	RT-qPCR/ELISA	Combined panel detects early CC	[29]
3	Zubillaga-Guerrero et al. (2020)	Mexico	Prospective	80	Liquid cytology	miR-16-1	RT-qPCR	Overexpression of miR-16-1 is associated with HR-HPV integration.	[26]
4	You et al. (2015)	China	Prospective	68	Plasma	miRNAs	RT-qPCR	miR-127 and miR-205 show diagnostic utility	[30]
5	Zhang et al. (2016)	China	Prospective	563	Serum	miRNAs	RT-qPCR	Noninvasive serum panel for diagnosis	[31]
6	Aftab et al. (2021)	Pakistan	Prospective	100	Urine	miRNAs	RT-qPCR	The urinary miRNA profile is a valuable noninvasive test.	[32]
7	Ning et al. (2021)	China	Prospective	380	Plasma	miRNAs	RT-qPCR	Six-miRNA panel identifies high-grade lesions.	[33]
8	Poinho et al. (2025)	Brazil	Prospective	39	Plasma	cfHPV-DNA	qPCR	Circulating cfHPV-DNA is associated with diagnosis and recurrence in CC.	[11]
9	Jeannot et al. (2016)	France	Retrospective	70	Serum	cfHPV-DNA	ddPCR	Circulating HPV DNA was detected in early-stage CC.	[34]
10	Jeannot et al. (2021)	France	Prospective cohort	94	Plasma	cfHPV-DNA	ddPCR	Post-treatment persistence predicts recurrence.	[35]
11	Sivars et al. (2022)	Sweden	Prospective cohort	54	Plasma	cfHPV-DNA	ddPCR	High levels correlate with worse survival.	[36]
12	Bonløkke et al. (2022)	Denmark	Retrospective	60	Plasma	cfHPV-DNA	ddPCR	cfHPV-DNA discriminates patients with advanced CC.	[37]
13	Bonløkke et al. (2024)	Denmark	Prospective cohort	179	Plasma	cfHPV-DNA	ddPCR + NGS	cfHPV-DNA predicts which patients require primary oncologic therapy.	[38]
14	Mittelstadt et al. (2023)	Germany	Prospective cohort	69	Plasma	cfHPV-DNA	NGS	cfHPV-DNA correlates with stage and recurrence.	[10]
15	Lalondrelle et al. (2023)	UK	Prospective	22	Plasma	cfHPV-DNA	NGS	cfHPV-DNA as an early predictor of CRT response.	[39]
16	Thangarajah et al. (2023)	Germany	Prospective	19	Plasma	cfHPV-DNA	ddPCR	Quantification of cfHPV-DNA is useful for monitoring.	[40]
17	Beaussire-Trouvay et al. (2024)	France	Prospective	97	Plasma	cfHPV-DNA	ddPCR	Prognostic value in locally advanced CC.	[41]
18	Bonin-Jacob et al. (2021)	Brazil	Prospective	410	Serum	Cytokines	ELISA	IL-6 and IL-10 are elevated in advanced stages.	[27]
19	Cai et al. (2022)	China	Prospective cohort	682	Serum	Cytokines	ELISA	Serum IL-6 predicts poor prognosis.	[42]
20	Vitkauskaite et al. (2024)	Lithuania	Cross-sectional	182	Serum	Cytokines	Multiplex	Cytokines are associated with reduced survival.	[43]
21	Domenici et al. (2021)	Italy	Prospective cohort	159	Whole blood-Tissue	Cytokines	IHC	Overexpression of IL-6 is associated with a poor prognosis.	[44]

^1^ Main features of the studies included in the systematic review. The table summarizes the study location, methodological design, sample size, type of biomarker investigated (e.g., circulating microRNAs, cfHPV-DNA, or serum cytokines), and platforms used for detection. The diversity of current research methodologies and the lack of standardization are reflected in the use of different techniques (e.g., RT-qPCR, ELISA, ddPCR, NGS) and in the varying number of patients analyzed.

## Data Availability

No new data were created or analyzed in this study. Data sharing is not applicable to this article.

## Supplementary Materials

The following supporting information can be downloaded at: https://www.mdpi.com/article/10.3390/ijms262110503/s1.

## Abbreviations

The following abbreviations are used in this manuscript:

cfHPV-DNA	Cell-free HPV DNA
miRNAs	microRNAs
ddPCR	droplet digital PCR
RT-qPCR	Reverse Transcription quantitative PCR
NGS	Next-Generation Sequencing
