# Supplementary material for: Liquid Biopsy Biomarkers for Cervical Cancer: A Systematic Review
Source: Int J Mol Sci. 2025 Oct 29;26(21):10503. doi: 10.3390/ijms262110503 (PMC12610596; doi:10.3390/ijms262110503)
Supplement: Supplementary file 1 [file ijms-26-10503-s001.zip › PROSPERO (2).pdf]

NIHR | National Institute for Health and Care Research

PROSPERO
International prospective register of systematic reviews

Liquid biopsy biomarkers in cervical cancer: a systematic review

Juan Carlos Bravata Alcantara, Enoc M Cortés Malagón

Citation
Juan Carlos Bravata Alcantara, Enoc M Cortés Malagón. Liquid biopsy biomarkers in cervical cancer: a systematic review. PROSPERO 2025 CRD420251150875. Available from https://www.crd.york.ac.uk/PROSPERO/view/CRD420251150875.

REVIEW TITLE AND BASIC DETAILS

Review title
Liquid biopsy biomarkers in cervical cancer: a systematic review

Condition or domain being studied
Cervical Cancer

Cervical cancer (including high-grade intraepithelial lesions and invasive disease).

Rationale for the review
Cervical cancer is still a major public health concern, especially in low- and middle-income nations where timely screening is frequently unavailable. Although there is no denying the usefulness of traditional methods like Pap smears and HPV DNA tests, they also have significant drawbacks, such as the potential to overlook early infections and the difficulty in differentiating between infections that resolve on their own and those that persist. Liquid biopsy biomarkers, such as serum cytokines, circulating microRNAs, and cfHPV-DNA, have gained attention recently as less invasive options that may be used for patient monitoring, diagnosis, and prognosis. Despite encouraging results, the lack of standardized methods has slowed their translation into everyday practice. To critically evaluate the available data and ascertain how these biomarkers might support clinical decision-making, a systematic review is required.

Review objectives
This review aims to understand how well liquid biopsy biomarkers—circulating microRNAs, cfHPV-DNA, and serum cytokines—perform in diagnosing and predicting the course of cervical cancer. It will also explore their potential role in disease monitoring and highlight the main methodological gaps that still need to be addressed before these tools can be used routinely in clinical practice.

Keywords
Cervical cancer; Liquid biopsy; Circulating microRNAs; cfHPV-DNA; Serum cytokines; Biomarkers; Molecular diagnostics

Country
Mexico

ELIGIBILITY CRITERIA

Population

Included
Women of any age with histologically confirmed high-grade cervical intraepithelial lesions (HSIL/CIN2+) or invasive cervical cancer (any FIGO stage), regardless of treatment status.

Excluded
Studies conducted in animals or cell lines; participants without histologically confirmed cervical lesions or cancer; case reports, narrative reviews, editorials, and conference abstracts; and studies lacking clinical outcomes or diagnostic accuracy data.

Intervention(s) or exposure(s)

Included
Eligible studies must evaluate at least one liquid biopsy biomarker (circulating miRNAs, cfHPV-DNA, or serum cytokines) in blood, urine, or cervical cytology samples from women with confirmed cervical intraepithelial neoplasia or cervical cancer. Studies should report diagnostic or prognostic performance, including sensitivity, specificity, predictive values, or correlation with clinical outcomes. Both single-biomarker and multi-biomarker panels will be considered.

Excluded

Studies that assess biomarkers exclusively in tissue samples, cell lines, or animal models; interventions not involving liquid biopsy biomarkers (e.g., imaging, tissue immunohistochemistry); studies without diagnostic accuracy, prognostic outcomes, or correlation with clinical endpoints; and narrative reviews, editorials, or conference abstracts.

Comparator(s) or control(s)

Included
Eligible comparators include healthy women without cervical intraepithelial lesions or cancer, histopathological confirmation (biopsy) as the diagnostic reference standard, and HPV DNA molecular testing. Studies must report comparator data that allow assessment of diagnostic or prognostic accuracy of liquid biopsy biomarkers.

Excluded
Studies lacking a defined comparator group; comparisons limited to animal or in vitro models; studies without histopathological confirmation, HPV testing, or appropriate clinical outcomes; and narrative reviews, case reports, or conference abstracts without primary comparator data.

Study design

Both randomized and nonrandomized study types will be included.

Included
Randomized clinical trials (if available) and non-randomized observational studies, including prospective and retrospective cohorts, case–control studies, and cross-sectional designs, that evaluate the diagnostic or prognostic accuracy of liquid biopsy biomarkers in cervical cancer.

Excluded
Case reports, narrative or systematic reviews, editorials, letters, conference abstracts, animal studies, and in vitro experiments using cell lines will be excluded. Studies without clinical outcomes or diagnostic accuracy data will also be excluded.

Context

This review focuses on clinical and research settings where liquid biopsy samples (plasma, serum, urine, or cervical cytology) are analyzed for biomarkers (miRNAs, cfHPV-DNA, cytokines) in women with high-grade cervical lesions or cervical cancer. Global evidence will be considered, with special attention to studies conducted in low- and middle-income countries such as Mexico and Latin America.

TIMELINE OF THE REVIEW

Date of first submission to PROSPERO

19 September 2025

Review timeline

Start date: 18 September 2025. End date: 31 December 2025.

Date of registration in PROSPERO

19 September 2025

AVAILABILITY OF FULL PROTOCOL

Availability of full protocol

A full protocol has not been written.

SEARCHING AND SCREENING

Search for unpublished studies

Only published studies will be sought.

Main bibliographic databases that will be searched

The main databases to be searched are *Embase - Embase via Ovid*, *LILACS - Latin American and Caribbean Health Sciences Literature*, *PubMed*, *SCI - Science Citation Index* and *Scopus*.

Search language restrictions

The review will only include studies published in English.

Search date restrictions

Databases will be searched for articles published from 1 January 2015 and before by 31 May 2025.

Other methods of identifying studies

Other studies will be identified by: *looking through all the articles that cite the papers included in the review ("snowballing" or forward citation searching)* and *reference list checking (backward citation searching)*.

Additional information about identifying studies

Reference list checking of included studies and forward citation searching (snowballing) will be performed to identify additional relevant articles not captured in the initial database search.

Link to search strategy

A full search strategy is available in the full protocol as described in the *Availability of full protocol* section

Selection process

Studies will be screened independently by at least two people (or person/machine combination) with a process to resolve differences.

Other relevant information about searching and screening

Two reviewers will independently screen titles, abstracts, and full texts using pre-specified inclusion and exclusion criteria. Discrepancies will be resolved by discussion and, if necessary, by consultation with a third reviewer. In addition to database searches, backward and forward citation tracking will be applied to ensure completeness.

DATA COLLECTION PROCESS

Data extraction from published articles and reports

Data will be extracted independently by at least two people (or person/machine combination) with a process to resolve differences.

Authors will be asked to provide any required data not available in published reports.

Study risk of bias or quality assessment

Risk of bias will be assessed using: *AMSTAR-2*, *Cochrane RoB-2* and *ROBINS-I*

Data will be assessed independently by at least two people (or person/machine combination) with a process to resolve differences.

Additional information will be sought from study investigators if required information is unclear or unavailable in the study publications/reports.

Reporting bias assessment

Risk of bias due to missing results will be assessed by evaluating potential publication and selective reporting biases. Funnel plot asymmetry and Egger's test will be applied when ≥10 studies are available for a given outcome. In addition, reported outcomes will be compared against study protocols or trial registry entries (when accessible) to detect selective outcome reporting.

Certainty assessment

Certainty of evidence will be assessed using the GRADE (Grading of Recommendations Assessment, Development and Evaluation) approach. Domains including risk of bias, inconsistency, indirectness, imprecision, and publication bias will be evaluated for each main outcome (e.g., sensitivity, specificity, prognostic performance). Evidence will be rated as high, moderate, low, or very low certainty, and summary of findings tables will be produced where applicable.

OUTCOMES TO BE ANALYSED

Main outcomes

Primary outcomes will include diagnostic accuracy measures of liquid biopsy biomarkers (miRNAs, cfHPV-DNA, and serum cytokines) for cervical cancer and high-grade intraepithelial lesions. Acceptable measures are sensitivity, specificity, positive predictive value, negative predictive value, and area under the ROC curve (AUC), as reported by each study. Prognostic outcomes will include recurrence, progression, overall survival, and disease-free survival. Outcomes may be measured at baseline, during treatment, or in post-treatment follow-up. Effect measures for synthesis will include pooled or narrative estimates of sensitivity, specificity, predictive values, hazard ratios, and relative risks, as appropriate.

Additional outcomes

Additional outcomes will include methodological and feasibility aspects of liquid biopsy biomarkers: (1) type of biological sample used (plasma, serum, urine, cervical cytology); (2) detection platform or assay employed (RT-qPCR, ddPCR, NGS, ELISA, multiplex); (3) cut-off thresholds or normalization strategies applied; (4) correlation with clinicopathological variables such as tumor stage, HPV genotype, or treatment modality. Outcomes will be reported at the time points available in each study. Effect measures will include descriptive statistics, correlation coefficients, and qualitative synthesis of methodological consistency or heterogeneity across studies.

PLANNED DATA SYNTHESIS

Strategy for data synthesis

No formal data synthesis is planned - data will be described but not combined.

CURRENT REVIEW STAGE

Stage of the review at this submission

Review stage	Started	Completed
Pilot work		
Formal searching/study identification		
Screening search results against inclusion criteria		
Data extraction or receipt of IPD		
Risk of bias/quality assessment		
Data synthesis		

Review status
The review is currently planned or ongoing.

Publication of review results
Results of the review will be published in English.

REVIEW AFFILIATION, FUNDING AND PEER REVIEW

Review team members
Dr Juan Carlos Bravata Alcantara (review guarantor and contact) ORCID: 0000-0002-3585-0051. Hospital Regional de Alta Especialidad Ixtapaluca. Mexico.

No conflict of interest declared.

Enoc M Cortés Malagón. ORCID: 0000-0002-6240-3468. Hospital Juárez de México. Mexico.

No conflict of interest declared.

Named contact
Dr Juan Carlos Bravata Alcantara (vaio_df@hotmail.com). ORCID: 0000-0002-3585-0051. Hospital Regional de Alta Especialidad Ixtapaluca. Mexico.

Review affiliation
Hospital Regional de Alta Especialidad de Ixtapaluca (HRAEI), Mexico

Funding source
Review has no specific/external funding but is supported by guarantor/review team (non-commercial) institutions.

Additional information about funding
No specific funding was required, apart from institutional access to databases and bibliographic software provided by HRAEI.

Peer review
There has been no peer review of this planned review.

ADDITIONAL INFORMATION

Additional information
Systematic review registered in PROSPERO on liquid biopsy biomarkers (miRNAs, cfHPV-DNA, and serum cytokines) in cervical cancer, aiming to provide clinical and public health evidence applicable to Latin America.

Review conflict of interest
Declared individual interests are recorded under team member details.. No additional interests are recorded for this review.

Medical Subject Headings
Biomarkers; Cytology; DNA; Female; Humans; Liquid Biopsy; MicroRNAs; Prognosis; Uterine Cervical Dysplasia; Uterine Cervical Neoplasms

SIMILAR REVIEWS

Check for similar records already in PROSPERO
PROSPERO identified a number of existing PROSPERO records that were similar to this one (last check made on 18 September 2025). These are shown below along with the reasons given by that the review team for the reviews being different and/or proceeding.

- liquid biopsy vs tissue biopsy for early cancer detection: A meta-analysis of diagnostic accuracy and clinical utility. [published 18 June 2025] [CRD420251073137]. The review was judged **not to be similar**
- Efficacy of Liquid Biopsy in detecting genetic mutations in lung cancer [published 26 August 2024] [CRD42024580227]. The review was judged **not to be similar**

- Circulating microRNAs as novel potential diagnostic biomarkers for cervical carcinogenesis: a systematic review [published 7 July 2021] [CRD42021259736]. The review was judged **not to be similar**
- The Role of Liquid Biopsy in Differentiating True Progression from Pseudoprogression and Radiation Necrosis in Glioblastoma: A Systematic Review [published 5 November 2024] [CRD42024606722]. The review was judged **not to be similar**
- Value of SOX1 Gene Methylation in Diagnosis of Cervical Cancer and Cervical intraepithelial neoplasia: A systematic review and Meta-Analysis [published 18 August 2024] [CRD42024577764]. The review was judged **not to be similar**

PROSPERO version history

- Version 1.0, published 19 Sep 2025

Disclaimer

The content of this record displays the information provided by the review team. PROSPERO does not peer review registration records or endorse their content.

PROSPERO accepts and posts the information provided in good faith; responsibility for record content rests with the review team. The guarantor for this record has affirmed that the information provided is truthful and that they understand that deliberate provision of inaccurate information may be construed as scientific misconduct.

PROSPERO does not accept any liability for the content provided in this record or for its use. Readers use the information provided in this record at their own risk.

Any enquiries about the record should be referred to the named review contact
