# Supplementary material for: Liquid Biopsy Biomarkers for Cervical Cancer: A Systematic Review
Source: Int J Mol Sci. 2025 Oct 29;26(21):10503. doi: 10.3390/ijms262110503 (PMC12610596; doi:10.3390/ijms262110503)
Supplement: Supplementary file 1 [file ijms-26-10503-s001.zip › S1.pdf]

PubMed search strategy:

(("cell-free DNA"[Title/Abstract] OR "cfDNA"[Title/Abstract] OR "circulating tumor DNA"[Title/Abstract] OR "ctDNA"[Title/Abstract] OR "plasma DNA"[Title/Abstract])

AND ("Human papillomavirus"[Mesh] OR "HPV"[Title/Abstract] OR "human papilloma virus"[Title/Abstract])

AND ("digital PCR"[Title/Abstract] OR "droplet digital PCR"[Title/Abstract] OR "ddPCR"[Title/Abstract])

AND ("sensitivity"[Title/Abstract] OR "specificity"[Title/Abstract] OR "diagnostic"[Title/Abstract] OR "detection"[Title/Abstract]))
